# Supplementary material for: Economic Uses of Salt-Tolerant Plants
Source: Plants (Basel). 2023 Jul 17;12(14):2669. doi: 10.3390/plants12142669 (PMC10385539; doi:10.3390/plants12142669)
Supplement: Supplementary file 1 [file plants-12-02669-s001.zip › plants-2492023-supplementary.pdf]

Supplementary Table

Supplementary Table S1 Categories of Economic Uses of salt-tolerant plants used in eHALOPH – based on the version of SEPASAL used by Aronson (1989).

**Code**	**Use**	**Sub Code**	**Use**				
000.0	FOOD AND DRINK						
						00001.0	Crop liste by FAO
		0100.0	Vegetables and fruit				
				0110.0	Vegetables		
						0115.1	Grain
				0120.0	Fruit		
				0190.0	Famine Food		
		0200.0	Beverages				
		0300.0	Cooking fats and oils				
				0310.0	Oilseed		
		0400.0	Miscellaneous food and drink				
				0410.0	Salt and salt substitutes		
		0500.0	Breeding stock				
1000.0	DOMESTIC PRODUCTS						
		1200.0	Soaps				
		1300.0	Cosmetics				
		1400.0	Dental				
		1700.0	Roofing thatching green roofs				
2000.0	TIMBER						
		2100.0	Fuel				

				2110.0	Fuelwood		
				2120.0	Charcoal		
				2130.0	Petroleum substitute; see also 8300.0 CHEMICALS		
		2400.0	Construction timber				
3000.0	FORAGE						
		3100.0	Grazing				
		3200.0	Browse				
		3300.0	Fodder				
4000.0	LAND USE						
		4400.0	Shade				
		4500.0	Soil stabilization				
				4510.0	Erosion control		
				4520.0	Sand stabilization		
		4600.0	Soil improvement				
		4700.0	Salt tolerance				
		4800.0	Ornamental				
				4850.0	Salt-tolerant ornamental		
5000.0	FIBERS						
		5200.0	Textiles				
6000.0	TOXINS	6000.0					
7000.0	MEDICAL						
		7100.0	General including traditional medicine				
				7160.0	Antibiotics		
				7200.0	Skin and mucosae		
				7500.0	Respiratory system		

				7600.0	Alimentary canal and digestive system		
				7800.0	Nervous system		
8000.0	CHEMICALS						
		8100.0	Carbohydrates				
		8200.0	Lipids, Essential oils				
		8300.0	Bioenergy/Biofuel				
